# Controlled Capture of Magnetic Nanoparticles from Microfluidic Flows by Ferromagnetic Antidot and Dot Nanostructures

**DOI:** 10.3390/nano15020132

**Published:** 2025-01-16

**Authors:** Reyne Dowling, Mikhail Kostylev

**Affiliations:** Department of Physics, The University of Western Australia, Perth, WA 6009, Australia; reyne.dowling@research.uwa.edu.au

**Keywords:** ferromagnetic materials, microfluidics, soft lithography, superparamagnetic iron oxide nanoparticles, magnetic nanoparticles, magnetophoresis, magnetic nanoparticle capture, magnetic nanoparticle trapping

## Abstract

The capture of magnetic nanoparticles (MNPs) is essential in the separation and detection of MNPs for applications such as magnetic biosensing. The sensitivity of magnetic biosensors inherently depends upon the distribution of captured MNPs within the sensing area. We previously demonstrated that the distribution of MNPs captured from evaporating droplets by ferromagnetic antidot nanostructures can be controlled via an external magnetic field. In this paper, we demonstrate the capture of magnetic nanoparticles from a microfluidic flow by four variants of antidot array nanostructures etched into 30 nm thick Permalloy films. The nanostructures were exposed to 130 nm MNP clusters passing through microfluidic channels with square cross-sections of 400 μm × 400 μm. In the presence of a parallel magnetic field, up to 83.1% of nanoparticles were captured inside the antidot holes. Significantly higher proportions of nanoparticles were captured within the antidots from the flow than when applying the nanoparticles via droplets. In the parallel field configuration, MNPs can be focused into the regularly spaced antidot indents in the nanostructure, which may be useful when detecting or observing MNPs and their conjugates. Conversely, up to 84% of MNPs were caught outside of antidots under a perpendicular magnetic field. Antidot nanostructures under this perpendicular configuration show potential for MNP filtration applications.

## 1. Introduction

### 1.1. Applications of Magnetic Nanoparticle Capture

Magnetic nanoparticles (MNPs) are indispensable to many nanotechnologies [1,2,3]. Most MNPs consist of iron oxide cores less than 20 nm in diameter coated in a protective layer, such as dextran. These particles display superparamagnetic properties and are considered to be composed of singular magnetic domains. When magnetized by an external magnetic field, these superparamagnetic nanoparticles can be finely manipulated for a number of applications, particularly in biosensing [4,5,6,7]. Magnetic biosensors employ magnetic nanoparticles as markers in magnetic nanoparticle assays designed to detect or quantify the presence of a particular target analyte, typically a protein or virus, in a sample such as water or saliva [8,9,10]. The assay sample is passed through a suspension of magnetic nanoparticles with coatings that bind the nanoparticles to the targets. The MNPs that have not been bound to a target analyte are usually separated from the bound MNPs via a magnetic separation technique [4,6,11,12]. The bound nanoparticles are then captured (or trapped) by a sensing surface or structure that produces an observable response in the presence of MNPs. The magnitude of this response is often proportional to the number of MNPs present in the sensing area and can be used to infer the quantity of the target analyte present in a sample. The dependence of the response upon the MNP quantity is indicative of the biosensor’s sensitivity, linearity, and limit of detection (LoD). Generally, the response also depends upon the locations of the captured nanoparticles relative to the sensing area [13,14]. The performance of a biosensor is therefore dependent upon the ability of the sensing area to capture MNPs.

Before discussing how magnetic nanoparticles are captured, it is important to understand how MNPs are detected and what structures are used for sensing. Many techniques have been developed for the detection of MNPs via fluorescent imaging or measurements of giant magnetoresistance (GMR), tunneling magnetoresistance (TMR), or the planar Hall effect (PHE) [9,12,15,16,17,18]. Magnetic nanoparticles can also be detected via the observation of relaxation in nuclear magnetic resonance (NMR) devices or superconducting quantum interference devices (SQUIDs), but these techniques typically do not require MNP capture [4,9,15,19,20]. Magnetoresistive sensors observe the nanoparticles indirectly through changes in the resistance of thin ferromagnetic multilayer spin-valves usually in the shape of flat, rectangular stripes. Many of these devices have successfully detected MNPs flowing through microfluidic channels passing over a sensing area [11,21,22,23,24,25,26]. In most of these devices, the sensing area is functionalized with biomolecules that bind to and capture the nanoparticles onto the surface of the multilayer sensor. Henriksen has shown that fluctuations in the distribution of nanoparticles on a flat magnetoresistive sensor can cause significant fluctuations in the sensor signal [13]. Their results emphasize the importance of understanding, predicting, and optimizing the capture of magnetic nanoparticles for improved sensor performance. Finally, the indirect detection of MNPs is possible via the observation of ferromagnetic resonance (FMR) in a ferromagnetic antidot nanostructure [14,27]. When the MNPs are caught inside the nanostructure, they produce noticeable changes in the nanostructure’s FMR. The location and number of the nanoparticles caught within this antidot nanostructure determines both the magnitude and polarity of the changes that occur in the FMR [14]. Controlling where the particles are captured is essential for the sensitive FMR-based detection of magnetic nanoparticles.

Another application requiring the capture of magnetic nanoparticles is in the profiling of rare cells for medical diagnostics and virology. As in biosensing, magnetic nanoparticles are selectively conjugated (or bound) to a particular species of cell or virus in a biological sample. The MNPs are then directed into traps via magnetophoresis, carrying the conjugated cells into the traps. With the cells immobilized within the traps, imaging can be performed to observe the morphology of each cell. For example, Ma et al. have demonstrated the profiling of circulating tumor cells (CTCs), employing a cobalt-based ferromagnetic nanostructure to direct, via magnetophoresis, MNP-tagged CTCs into a trap [28]. Ideally, the cells are captured in an ordered arrangement that allows for the easy imaging and identification of the cells. As such, the capture of MNPs into an ordered array of antidots may be of interest for cell profiling applications. Naturally, the capture efficiency for such a nanostructure must be almost 100% to ensure that the conjugated MNPs are captured only within the antidots.

Magnetophoresis and magnetic nanoparticle capture are also employed to separate or filter MNPs conjugated to targets such as biomolecules, viruses, and small cells from a liquid sample (including saliva, blood, and urine). The sample flows into or over a magnetic nanostructure that alters the trajectory of each nanoparticle via magnetophoresis. In the case of magnetic separation, the flows of conjugated and non-conjugated MNPs are separated due to the difference between their hydrodynamic volumes [6,11,29]. Magnetic separation is often performed prior to magnetic nanoparticle biosensing, as the presence of non-conjugated MNPs may produce false-positive detections of the target. Kokkinis et al. demonstrated the separation of MNPs conjugated to *Escherichia coli* bacteria, via a series of conductive microstructures, and their detection via a GMR sensor [11]. In the case of magnetic filtration, both conjugated and non-conjugated MNPs are attracted towards and captured by the nanostructure, removing them from the flow. For example, Zarutskaya and Shapiro have investigated the use of a series of sieves consisting of a fibrous ferromagnetic nanostructure for the attraction and capture of MNPs from a microfluidic flow [30]. This fibrous nanostructure could be substituted for one of the antidot or dot array nanostructures investigated in this research. For such a magnetic nanoparticle filter, the capture efficiency should be almost 0% to ensure no MNPs flow through the nanostructure.

### 1.2. Magnetophoresis of Magnetic Nanoparticles

Magnetophoresis refers to the movement of magnets due to the magnetic forces that occur between magnetic dipoles and their associated magnetic fields. Magnetophoresis is primarily discussed in the context of magnetic particles in a liquid medium, in which the presence of an external magnetic field gradient exerts an unbalanced magnetic force—known as a magnetophoretic force—upon the particles, causing them to travel along the magnetic field gradient. The magnetophoresis of magnet particles will occur in any system containing a non-uniform magnetic field, including magnetic tweezers and most magnetic particle sensors. Magnetophoresis also occurs within the suspension of magnetic particles itself, attracting nearby particles together to form unwanted clusters or chains in a process known as agglomeration or flocculation [31]. The occurrence of magnetic particle agglomeration increases in the presence of an external magnetic field, since the magnetophoretic forces between particles are stronger. As such, magnetophoresis should be considered whenever magnetic particles are exposed to external magnetic fields. However, the motion of nanoscale magnetic particles in a liquid solution is usually dominated by Brownian and viscous drag forces due to the particles’ small hydrodynamic volumes [32].

Magnetic field gradients are often employed to direct the motion of MNPs via magnetophoresis and collect them in desired locations [24,29,33,34,35]. For example, Cardoso employed a neodymium magnet to separate MNPs from a suspension flowing through a series of microfluidic channels [36]. This principle is also used to remove microbes in water treatment systems [37]. To be precise, magnetophoresis draws the MNPs along the gradient of the squared magnitude of the total magnetic field applied to the MNP [32,38]. This total magnetic field is a combination of any external magnetic fields present and any stray magnetic fields produced by nearby magnetic objects, such as other MNPs and ferromagnetic nanostructures. The magnetophoretic force ${\vec{F}}_{m}$ that is exerted onto each magnetic nanoparticle by the total magnetic field $\vec{B}$ follows the equation [34,39]

(1) $${\vec{F}}_{m}=A\vec{\nabla }{\left|\vec{B}\right|}^{2},$$

where the constant A is defined by

(2) $$A=\frac{V_{mnp}\left(\chi_{mnp}-\chi_{fluid}\right)}{2\mu_{0}}.$$

In these equations, $V_{mnp}$ is the volume of the nanoparticle, $\chi_{mnp}$ is the magnetic susceptibility of the nanoparticle, $\chi_{fluid}$ is the magnetic susceptibility of the water, and $\mu_{0}$ is the vacuum magnetic permeability.

Many magnetic biosensors also use magnetophoresis to direct and capture the nanoparticles onto the sensing areas. The magnetophoretic forces can be generated by a constant, but non-uniform, external magnetic field, such as that of a permanent magnet, or by a temporary magnetic field generated by an electromagnet, depending upon the requirements of the device [18,40,41]. For instance, Little et al. used a constant external magnetic field to generate magnetic fields on the periphery of four spinvalves used for sensing [23]. Alternatively, Kokkinis et al. employed a sequence of microscopic conductive stripes as electromagnets to direct MNPs onto a sensor [11]. Naturally, the magnetophoretic forces present in every MNP sensing device will be different. The magnetophoretic forces must be modelled to determine the ideal magnetic fields that must be applied for efficient nanoparticle capturing. This study will focus on the influence of a static external magnetic field upon the capturing of MNPs inside the ferromagnetic antidot nanostructures of Metaxas et al. and Sushruth et al. [14,27]

### 1.3. Capture of Magnetic Nanoparticles by Antidot Nanostructures

**Figure 1:**
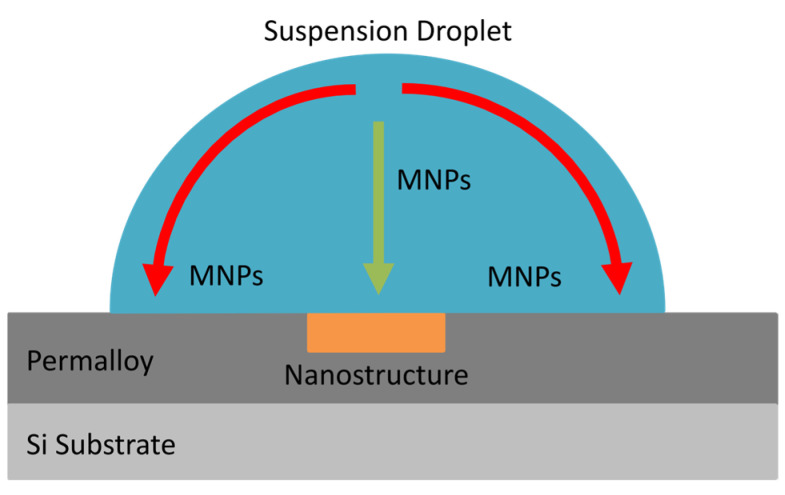
A droplet containing a suspension of magnetic nanoparticles placed on the surface of a Permalloy film and centered over a nanostructure etched into the film’s surface. The green and red arrows indicate the movement of MNPs under magnetophoretic and capillary forces, respectively.

**Figure 2:**
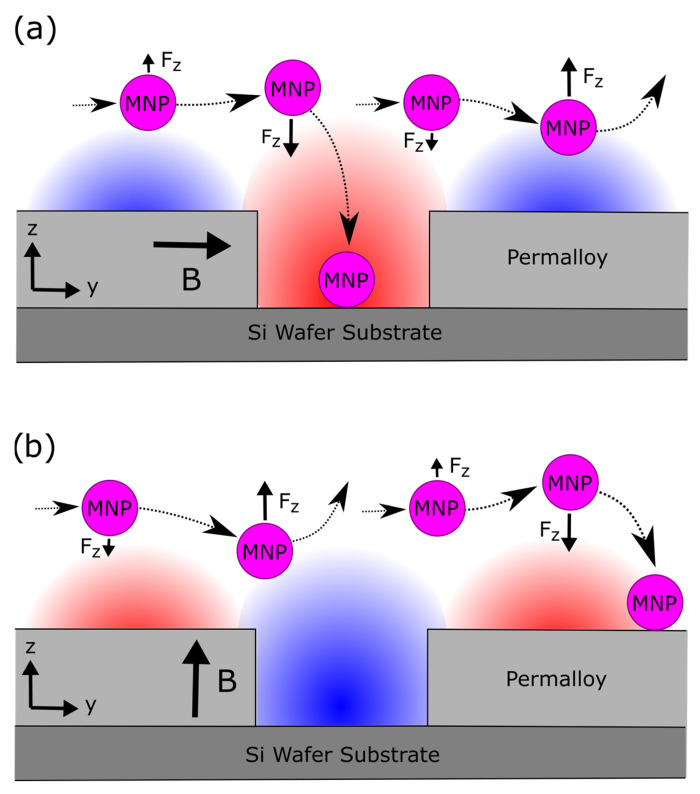
A simplified depiction of the attractive component of the magnetophoretic force (F_z_) acting upon nanoparticles moving past an antidot etched into a Permalloy film in the presence of (**a**) a parallel external magnetic field and (**b**) a perpendicular external magnetic field. Regions of attractive and repulsive magnetophoretic forces are indicated in red and blue, respectively.

In their experiments with FMR-based magnetic nanoparticle detection, Sushruth applied droplets of MNP suspensions onto the surface of 30 nm thick Permalloy films containing antidot nanostructures—square arrays of 300 nm diameter circular holes [14]. The particles were caught on the nanostructures as the droplets evaporated. The non-uniformity of the ferromagnetic nanostructure’s local magnetic field generated weak magnetophoretic forces that attracted some of the MNPs towards the nanostructure. As the droplet dried, outwards, capillary flows within the droplet also directed many MNPs towards the droplet’s surface, resulting in a ‘coffee ring’ of agglomerated nanoparticles on the surface of the film once the droplet dried [42]. A simplified depiction of the motion of MNPs within an evaporating droplet is presented in Figure 1. In a previous study, we demonstrated both experimentally and computationally that an external magnetic field can be used to control and enhance the capture of MNPs from droplets deposited onto a ferromagnetic antidot nanostructure [43]. Due to the complexity of the ferromagnetic nanostructures, the micromagnetic modeling of the nanostructures was performed numerically via MuMax3. Our modeling indicated that an external magnetic field generates stronger magnetophoretic forces that vary across the surface of the nanostructure [43,44,45]. The experiments showed that a magnetic field applied parallel to the surface of the nanostructure promotes the capture of MNPs inside of the antidot holes. Conversely, a magnetic field applied perpendicular to the surface of the nanostructure promotes capture of MNPs outside of the antidots. The magnetophoretic force acting upon MNPs moving past a ferromagnetic nanostructure in the presence of each external magnetic field are depicted in Figure 2. The application of a magnetic field in either direction also prevented MNP ‘coffee ring’ agglomerations from forming around the edges of the droplets [42,43].

Unlike most biosensors, Sushruth’s ferromagnetic detectors did not utilize microfluidics or MNP separation, and instead, required the dispersion of MNPs to be applied to the devices manually using pipettes. Sushruth’s technique could be improved through integration with microfluidics, in which case the nanoparticles would be captured by the antidot nanostructure while flowing through a microfluidic channel. Microfluidics is complementary to biosensing and the integration of the two has many advantages, such as reduced sample volumes, capability for multiplexed sensing, and integration with complex microfluidic systems, including MNP separation systems [21,22,25]. Multiple studies have been performed on the capture of flowing magnetic nanoparticles by other nanostructures. For example, Ezzaier et al. demonstrated that the distribution of magnetic nanoparticles captured by an array of magnetized micropillars is highly dependent upon the orientation of an external magnetic field with respect to the flow and nearly independent of the array geometry [46].

**Figure 3:**
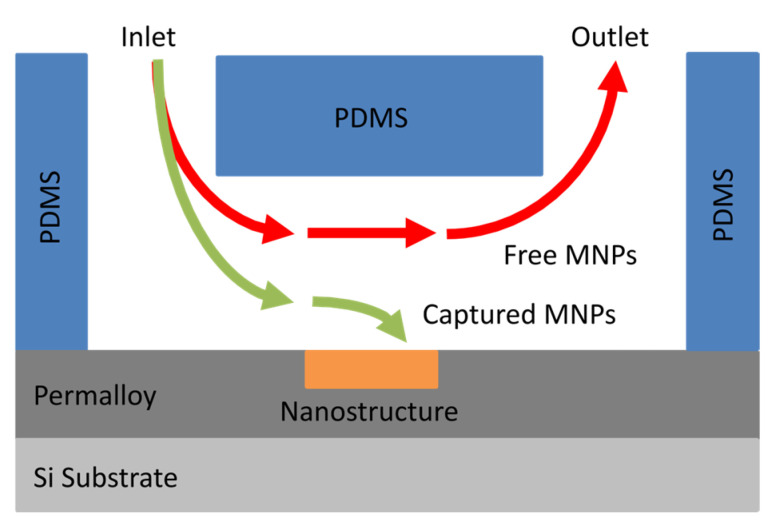
Magnetic nanoparticles passing through a PDMS microfluidic channel centered over a nanostructure etched into a Permalloy film. The MNPs that pass close to the nanostructure are attracted towards and captured by the nanostructure, while the remaining MNPs continue to flow through the channel.

However, to date, no studies have investigated the capture of flowing magnetic nanoparticles by ferromagnetic antidot array nanostructures. This study aims to fill this gap in knowledge, as the capture of MNPs is a vital parameter to consider in the design of FMR-based magnetic biosensors. To this end, microfluidic channels were fabricated from polydimethylsiloxane (PDMS) using a soft lithographic technique. Nanostructures were etched into thin Permalloy films via focused ion beam (FIB) lithography before each film was sealed to a PDMS microfluidic film. A suspension of MNPs was passed though the microfluidic channels under parallel and perpendicular external magnetic fields. As shown in Figure 3, the magnetophoretic forces produced by the ferromagnetic nanostructure attract nearby MNPs towards the nanostructure. These nanoparticles are then captured when they come into contact with the surface. However, nanoparticles far from the nanostructure are not attracted and continue flowing into the outlet of the channel. The resulting distributions of nanoparticles caught within the nanostructures were analyzed and compared to the previous results obtained with droplets. The nanostructure capture in microfluidic flow conditions was qualitatively similar to capture with droplets evaporating on the surface of the structure. However, the difference between the parallel and perpendicular magnetic fields was far stronger when capturing from a flow. In particular, the parallel magnetic field captured far more nanoparticles within the antidots than the application of a perpendicular magnetic field. The parallel field also demonstrated a higher capture efficiency than for the droplet arrangement, suggesting that microfluidic flow may improve the capture efficiency. These results will be helpful in designing nanostructures that more effectively capture MNPs in future FMR-based magnetic nanoparticle biosensors.

## 2. Materials and Methods

### 2.1. Fabrication of Ferromagnetic Antidot Nanostructures

**Figure 4:**
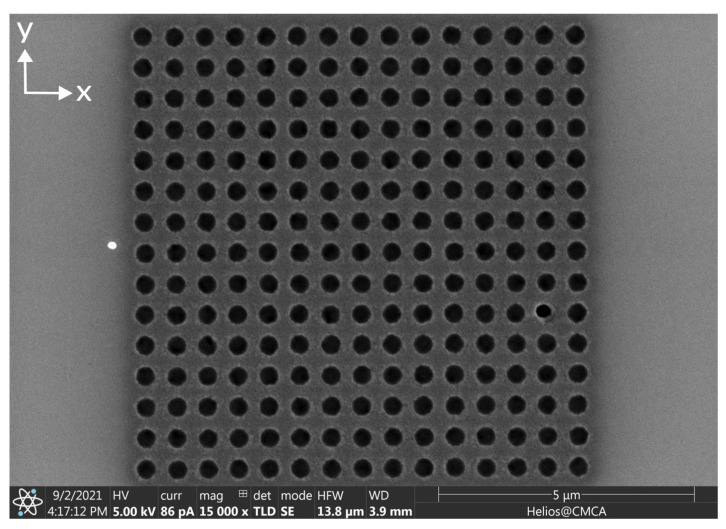
SEM image of an array of 400 nm diameter circular antidots etched into 30 nm thick Permalloy film using a focused ion beam. This image was obtained before the application of any MNPs. The centers of the antidots are separated by 600 nm in both directions.

Three ferromagnetic antidot nanostructures were fabricated from Permalloy thin films using focused ion beam lithography. First, a 30 nm layer of Permalloy (${Ni}_{80}{Fe}_{20}$) was coated onto three separate $5\times 5\;{mm}^{2}$ silicon wafers using magnetron sputtering in an argon atmosphere. The Permalloy layers were magnetron-sputtered from a Permalloy target onto the silicon substrates at a temperature of 150 °C and the argon pressure was kept to 6 mTorr. The base pressure in the chamber was lower than $10^{-7}\;Torr$. The continuous Permalloy films were then etched using a focused ion beam (FEI Helios, Thermo Fisher Scientific Inc., Waltham, MA, USA) [47,48,49,50,51,52,53]. The beam current was set to 80 pA and the accelerating voltage was 30 kV. The beam etched 15 × 15 square arrays of circular antidots with 400 nm diameter and 600 nm separation between the centers of the antidots, as shown in Figure 4. To explore the statistics of MNP capture in the antidots, the etching was repeated to obtain at least 20 separate circular antidot arrays on each of the three films. To investigate the influence of the array geometry on nanoparticle capture, the ion beam also etched at least 20 arrays each of circular dots (the inverse of an antidot nanostructure), square antidots, and square dots. An SEM image for each of these three nanostructure variants can be found in Appendix A. In total, each Permalloy film contained approximately 80 separate nanostructures.

### 2.2. Fabrication of Microfluidic Channels

**Figure 5:**
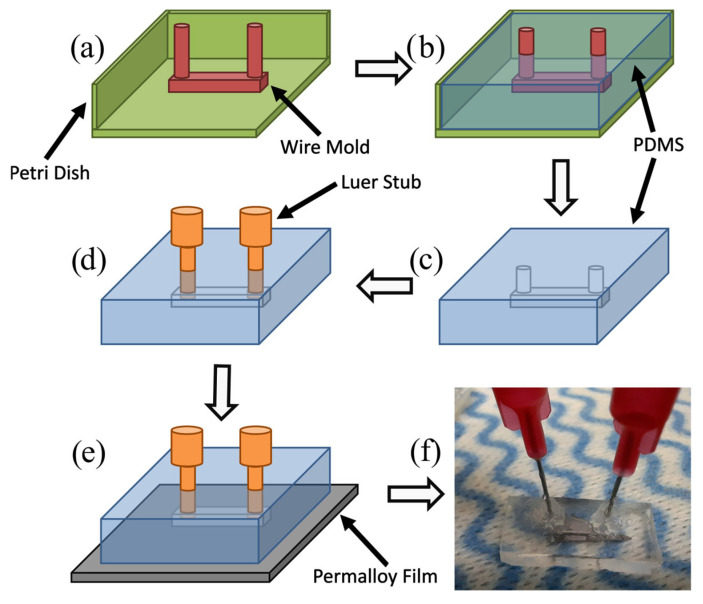
The first soft lithographic process used to fabricate microfluidic channels: (**a**) a mold is created using 0.4 mm diameter wire (red) and placed inside a container such as a Petri dish (green), (**b**) PDMS elastomer (blue) is poured into the mold, and (**c**) the PDMS sets over 24 h and is removed from the container. The wire mold is removed from the PDMS film using tweezers; (**d**) Luer stubs (orange) are inserted into the slots at both ends of the channel to form inlet and outlet ports. (**e**) The PDMS film is pressed against the surface of a silicon wafer or nanostructured Permalloy film to seal the microfluidic channel closed. (**f**) A photograph of the resulting microfluidic channel pressed against the surface of a 30 nm thick Permalloy film.

Microfluidic channels of dimensions $0.4\times 0.4\times 10\;{mm}^{3}$ were fabricated using the soft lithography process shown in Figure 5 [54,55,56]. Using this method, one can avoid the relatively expensive and complex processes associated with hard lithography. Since complex molds can be created in only a few minutes, this process is particularly useful for fabricating prototype microfluidic devices. These microchannel molds could also be 3D-printed for similar results with greater repeatability. A mixture of the PDMS elastomer base (Sylgard^®^ 184 silicone elastomer, Dow Chemical Company, Midland, MI, USA) and curing agent were poured over the molds in a ratio of 10:1 by volume and left to set for 24 h in ambient laboratory conditions. Luer stubs (Instech Laboratories, Inc., Plymouth Meeting, PA, USA) were inserted into the ends of the channels, which act as inlets and outlets, for connecting the external tubing. The PDMS films were then pressed onto the surfaces of the Permalloy films, creating a water-tight seal between the two. An optical microscope was used to position the channels so that the nanostructures were located at the centers of the microchannels.

### 2.3. Preparation of Magnetic Nanoparticles

The inlets of the microchannels were connected to syringes slotted onto a syringe pump (Harvard Apparatus Pump 11 Elite, Harvard Apparatus, Holliston, MA, USA). Each syringe was filled with 3 mL of 0.1 mg/mL suspensions of dextran-coated, 130 nm iron oxide magnetic nanoparticle clusters (Nanomag^®^-D, micromod Partikeltechnologie GmbH, Rostock, Germany). The concentration of the nanoparticle suspension was originally 25 g/mL, which was diluted to 0.1 mg/mL using distilled water. The relatively low concentration was chosen to prevent the nanostructures from being completely covered by nanoparticles and to avoid the formation of MNP agglomerates and chains that occur in dense MNP suspensions under an applied magnetic field [29,31,57,58,59]. The channel outlets were connected via tubing to glass beakers serving as reservoirs.

### 2.4. Capture of Magnetic Nanoparticles

**Figure 6:**
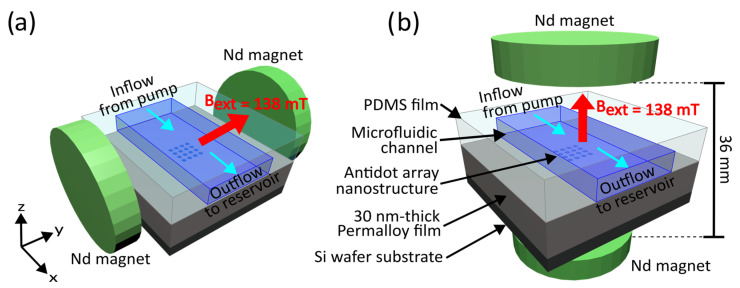
The two configurations of neodymium permanent magnets producing a static magnetic field either parallel or perpendicular to the Permalloy films. In both cases, the Permalloy film is placed equidistant from two cylindrical magnets connected to iron yokes, which provide a uniform magnetic field with a strength of 138 mT (1.38 kG) at the location of the nanostructures. In diagram (**a**), the magnetic field is directed along the x-axis, parallel to the Permalloy film. In diagram (**b**), the magnetic field is directed along the z-axis, perpendicular to the Permalloy film. Both diagrams are not to scale.

The first nanopatterned Permalloy film was placed between two cylindrical neodymium magnets applying an external magnetic field aligned parallel with the plane of the Permalloy film, as shown in Figure 6a. The two magnets were separated by 36 mm and attached to cylindrical iron yokes to increase the strength and uniformity of the applied magnetic field. At the location of the nanostructures etched into the Permalloy film, equidistant from both magnets, the applied magnetic field was measured to be 138 mT (1.38 kG). This experiment was repeated with a second nanopatterned Permalloy film placed between the two cylindrical magnets, with the magnetic field now directed perpendicularly to the plane of the film, as illustrated in Figure 6b. The nanostructures were positioned at the same location, where the applied magnetic field was 138 mT (1.38 kG). The third nanopatterned Permalloy film was not placed inside an external magnetic field. The syringe pump was then activated and infused the MNPs through each of the microfluidic channels at a rate of 1 mL/min. Afterwards, 3 mL of distilled water was pumped through each channel to flush the remaining MNPs out of channels before they could settle, and another 3 mL of air was pumped through the channels to dry them. Once the infusions were complete, the PDMS films were peeled off of the Permalloy films and the nanostructures were imaged using a scanning electron microscope (FEI Verios).

### 2.5. Analysis of Captured Magnetic Nanoparticles

**Figure 7:**
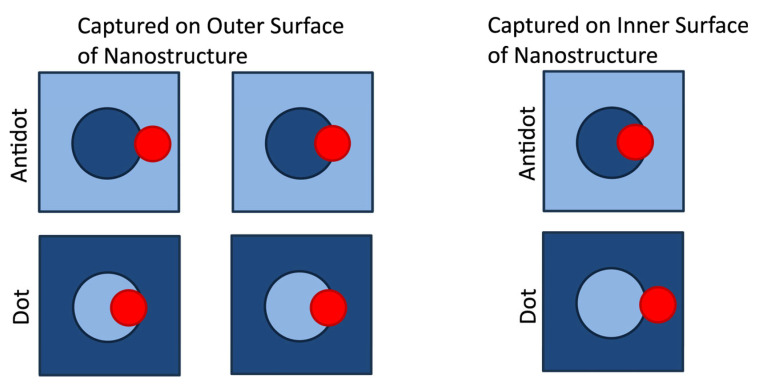
Diagrams depicting the position of a magnetic nanoparticle (red) with respect to an antidot or dot, indicating whether these nanoparticles are considered to have been trapped on the outer or inner surface of the nanostructure. Lighter blue regions represent areas containing Permalloy, while darker regions represent empty spaces in which Permalloy has been removed from the film.

In analyzing the microscope images, the nanoparticles were counted and sorted as captured inside or outside of each nanostructure, following the criteria illustrated in Figure 7. Particles trapped ‘inside’ of the nanostructure are caught on the internal surfaces of the nanostructure. Conversely, particles trapped ‘outside’ of the nanostructure are caught on the outer surface of the structure. For a nanoparticle to be considered as trapped inside of the nanostructure, most of the nanoparticle must be located inside of the nanostructure. Otherwise, the particle is counted as trapped on the outside of the nanostructure. Large agglomerations of more than three connected nanoparticles were not counted. The counting results were then used to calculate the average number of nanoparticles captured inside of each nanostructure geometry, $N_{IN}$, and the average number of nanoparticle caught outside of each nanostructure geometry, $N_{OUT}$. Nanostructures with dot geometries contain greater volumes of empty space for capturing magnetic nanoparticles internally than their corresponding antidot geometries. This difference between the volume of empty space, $V_{EMPTY}$, and the volume of magnetic material in the nanostructure, $V_{NANO}$, is accounted for by scaling the average numbers of particles inside, $N_{IN}$, and particles outside, $N_{OUT}$, by the scale factors $S_{IN}$ and $S_{OUT}$, respectively, where

(3) $$S_{IN}=\frac{V_{NANO}}{V_{EMPTY}},$$

and

(4) $$S_{OUT}=\frac{V_{EMPTY}}{V_{NANO}},$$

Finally, the capture efficiency of each array was calculated using Equation (5) below. This efficiency is indicative of the ability of each variation in the nanostructure geometry to capture MNPs internally and enables the direct comparison of the four geometries. The capture efficiency, $C_{EFF}$, estimates the proportion of MNPs that are or could be captured inside of each nanostructure geometry in comparison to the total number of nanoparticles captured by the nanostructure (both internally and externally):

(5) $$C_{EFF}=\frac{S_{IN}N_{IN}}{S_{IN}N_{IN}+S_{OUT}N_{OUT}}.$$

A nanostructure’s capture efficiency is high ($C_{EFF}>0.5)$ when most of the captured MNPs have been attracted into the empty spaces of the nanostructure. This will occur when most of the space within and surrounding the empty regions of the nanostructure contains attractive magnetophoretic force. Most of the remaining space surrounding regions of Permalloy should contain repulsive magnetophoretic force to encourage the movement of MNPs into regions of attractive magnetophoretic force. This arrangement is illustrated in Figure 2a. The capture efficiency increases when magnetophoresis directs a greater proportion of MNPs into the nanostructure. Conversely, with a low capture efficiency ($C_{EFF}$ < 0.5), most of the captured MNPs have been attracted onto the outer surface of the Permalloy film (not in the empty space within the nanostructure). This will occur when the space surrounding the empty regions of the nanostructure contains mostly repulsive magnetophoretic force, while the remaining space surrounding regions of Permalloy should contain attractive magnetophoretic force. This arrangement is illustrated in Figure 2b. The geometry of the nanostructure and the external magnetic field both determine the magnetophoretic force exerted by the nanostructure upon magnetic nanoparticles and, by association, the capture efficiency of the nanostructure.

## 3. Results

### 3.1. Results—MNP Capture Without External Magnetic Field

**Figure 8:**
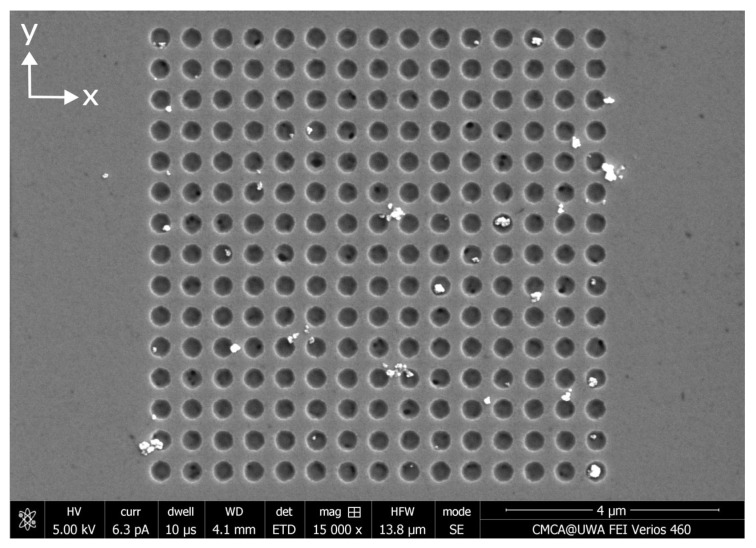
SEM image of a distribution of MNPs captured by an array of circular antidots etched into a thin Permalloy film. The MNPs were applied to this nanostructure via a microfluidic flow in the absence of any external magnetic fields.

After the infusion of magnetic nanoparticles through the microfluidic channel, the distributions of particles caught by each of the 80 nanostructures were imaged by scanning electron microscopy. The nanoparticles were then counted and the results were analyzed according to the method outlined in Section 2.5. The SEM image in Figure 8 displays one example of the distribution of magnetic nanoparticles captured by an array of circular antidots in the absence of an external magnetic field. SEM images showing MNPs captured by the remaining three nanostructure variants in the absence of an external magnetic field can be found in Appendix B. To aid in observing the distributions of captured MNPs, recolored variations are provided in Appendix E of the SEM images for the MNP distributions captured by the circular antidots and dot nanostructures. The distributions are relatively uniform across the antidot and dot arrays, with particles captured both inside and outside of the antidot holes. The nanoparticle distributions obtained from MNPs caught from a flow are qualitatively similar to those obtained when the MNPs were deposited onto the nanostructures in droplets. This is reflected in the total and average number of particles counted for each of the four nanostructure geometries, which is tabulated in Table 1 below. These values were weighted according to Equations (3) and (4) to account for the differences in the total volumes of the empty space available for each nanostructure variant. The capture efficiency for each variant of the nanostructure geometry is also included and was calculated using Equation (5). Table 1 also includes the capture efficiencies obtained from droplet depositions performed in our previous study on MNP capture via ferromagnetic nanostructures [43].

As with the distributions obtained from droplets onto the surface of the nanostructures, the antidot geometries show stronger capture efficiencies than the dot geometries. In addition, the circular antidot nanostructures again showed the strongest capture efficiency of 62%, which is lower than the 70.8% obtained from deposition by droplets. However, the other three geometries show significantly lower capture efficiencies than those obtained from droplet depositions. In particular, the efficiency of the square antidot geometry fell from 55.9% to only 29.6%. This indicates that depositing the nanoparticles via a microfluidic flow decreases the ratio of nanoparticles caught inside the empty regions of the nanostructures rather than increasing it. The combined average numbers of MNPs captured by each nanostructure (both inside and outside) are much higher than in the droplet case (~60–70 MNPs), as a larger sample volume was required to pump the MNP suspension through each microfluidic channel. The square dot nanostructure variant caught the greatest number of MNPs, with a total of 545 MNPs captured across 21 nanostructures. However, the circular dot nanostructures captured only 13 MNPs across 20 nanostructures, averaging only a single MNP captured by each nanostructure. This is significantly fewer MNPs captured when introducing the nanoparticles via droplets, in which case the circle dot nanostructures captured an average of 10 MNPs per nanostructure [43].

### 3.2. Results—MNP Capture with External Magnetic Field Parallel to Nanostructure

**Figure 9:**
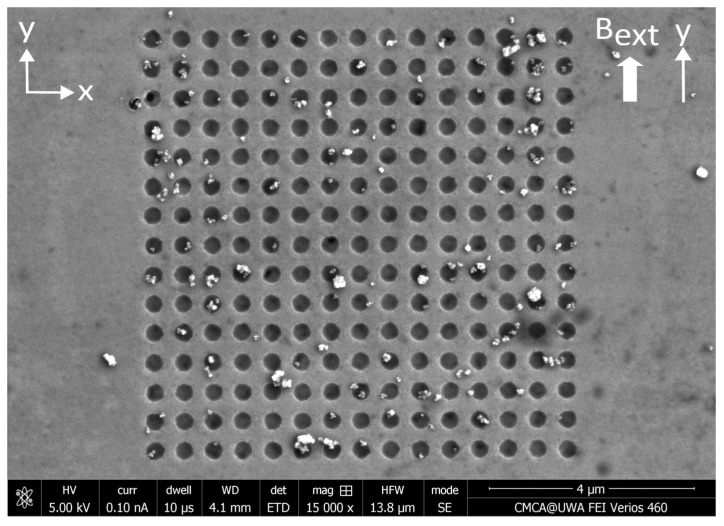
SEM image of a distribution of MNPs captured by an array of circular antidots etched into a thin Permalloy film. The MNPs were applied to this nanostructure via a microfluidic flow under a 138 mT (1.38 kG) static magnetic field applied parallel to the plane of the nanostructure, along the y-axis.

The SEM image in Figure 9 displays one example of the distribution of MNPs captured by an array of circular antidots under an external magnetic field directed parallel to the Permalloy film. SEM images showing MNPs captured by the remaining three nanostructure variants in the presence of a parallel external magnetic field can be found in Appendix C. The total count of MNPs captured inside and outside of each of the four nanostructure variants is collected in Table 2, along with the capture efficiencies of each geometry. The images and capture efficiencies indicate that almost half, or more, of the nanoparticles are caught within the empty spaces of the nanostructure. In particular, the circle antidots exhibited the highest capture efficiency of 83.1%—more than the highest efficiency obtained, also in circle antidots, of 70.5% when the MNPs are applied via droplets. The lowest capture efficiency among all four variants was again achieved by the circle dot nanostructures. This capture efficiency was 43.9%—only 0.2% lower than the 44.1% observed when MNPs are applied via droplets. The application of an external magnetic field strengthened the magnetophoretic forces between each nanoparticle and noticeably increased the occurrence of MNP agglomeration. Large agglomerations of MNPs were not counted and did not contribute to the results presented in Table 2.

The application of a parallel magnetic field significantly increased the ratio of MNPs captured inside of the empty spaces within the nanostructures. The greatest rise in the capture efficiency was observed in the circle and square antidot nanostructure variants. For example, upon the addition of a static magnetic field parallel to the plane of the Permalloy film, the capture efficiency of the square antidot nanostructures increased from 29.6% to 79.3%. In addition, the total number of MNPs caught by each of the four nanostructure variants in the presence of a magnetic field parallel to the nanostructures was far greater than the numbers of MNPs captured in the absence of an external magnetic field. The square dot variation once again captured the most nanoparticles, with a total of 1625 MNPs captured—198% more MNPs than the total of 545 MNPs obtained in the absence of an external magnetic field.

### 3.3. Results—MNP Capture with External Magnetic Field Perpendicular to Nanostructure

**Figure 10:**
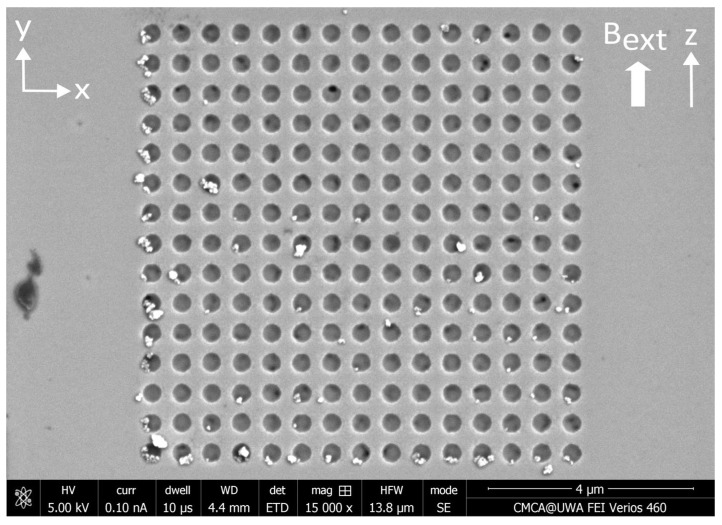
SEM image of a distribution of MNPs captured by an array of circular antidots etched into a thin Permalloy film. The MNPs were applied to this nanostructure via a microfluidic flow under a 138 mT (1.38 kG) static magnetic field applied perpendicular to the plane of the film along the z-axis.

The SEM image in Figure 10 displays one example of the distribution of MNPs captured from the microfluidic flow by an array of circular antidots in the presence of a perpendicular external magnetic field. SEM images showing MNPs captured by the remaining three nanostructure variants in the presence of a perpendicular external magnetic field can be found in Appendix D. The results of counting the nanoparticles captured by the four nanostructure variants are summarized in Table 3. When applying the MNPs to the nanostructures via droplets, the capture efficiencies were found to be particularly low when a magnetic field was applied perpendicular to the plane of the Permalloy film. For this configuration, the highest capture efficiency was observed in the square antidot nanostructures at only 28.8%. In addition, the circle dot geometry displayed the lowest efficiency of only 7.5%, significantly lower than the other three variants. When the nanoparticles are applied via a microfluidic channel rather than in droplets, the capture efficiencies are noticeably higher. With microfluidics, the highest capture efficiency of 77% was obtained from the circle antidot nanostructures. This value is considerably larger than the value obtained when applying the MNPs via droplets and is comparable to the largest capture efficiency observed in circle antidots of 83.1% obtained with microfluidics under the application of a parallel magnetic field.

However, the other three geometries exhibit much lower capture efficiencies under a perpendicular magnetic field than in a parallel field. This was expected, since simulations have shown that a perpendicular magnetic field results in repulsive magnetophoretic forces above the empty spaces in a nanostructure and attractive magnetophoretic forces above the regions containing Permalloy [43]. This arrangement of the magnetophoretic force directs the nanoparticles onto the Permalloy regions rather than between them, reducing the capture efficiency. In addition, far fewer MNPs were captured from the microfluidic flow in the presence of a perpendicular external magnetic field than in the presence of a parallel external magnetic field. The circle dot nanostructure variant captured the most MNPs, with 723 MNPs captured across 23 nanostructures. Still, the application of a perpendicular magnetic field resulted in significantly more nanoparticles being captured from the microfluidic flow than in the arrangement without a magnetic field (545 MNPs captured by square dot nanostructures).

One can also observe that many MNPs were caught along two connected edges of the nanostructure, but only when the static magnetic field was perpendicular to the film. This phenomenon was also observed in our previous experiments on MNP capture by ferromagnetic nanostructures in which the MNPs were applied via droplets [43]. The numerical modelling of a ferromagnetic antidot nanostructure suggested that this phenomenon is a result of the 138 mT (1.38 kG) perpendicular magnetic field being too weak to completely saturate the ferromagnetic nanostructure, creating regions of attractive magnetophoretic forces along two edges of the nanostructure [43].

## 4. Discussion

### 4.1. Discussion—MNP Capture Without External Magnetic Field

Introducing magnetic nanoparticles to the ferromagnetic nanostructures via a microfluidic channel, without applying a magnetic field, was observed to decrease the capture efficiency of the antidot nanostructures, rather than increasing it. This may be a result of the hydrodynamic forces dominating the magnetophoretic forces in the absence of an external magnetic field, as the capture efficiencies were higher when introducing the MNPs via microfluidics in the presence of an external magnetic field. Further investigation is required to better understand the hydrodynamics of an MNP flowing over an antidot nanostructure. In addition, fewer MNPs were captured by the nanostructures when applied via microfluidic channel than when the MNPs are applied to the nanostructures via droplets. Since the magnetic field gradient, and resulting magnetophoretic force, produced by the ferromagnetic nanostructure in the absence of an external magnetic field was weak, most of the nanoparticles in the suspension simply flowed past the nanostructures. Only the few nanoparticles that travelled close to the surface of the nanostructure experienced magnetophoretic forces strong enough to attract them towards and onto the nanostructures. A simplified illustration of the flows of magnetic nanoparticles through a microfluidic channel is depicted in Figure 3. Increasing the number of MNPs captured by the nanostructures requires the strengthening of the magnetophoretic force acting upon each MNP. As observed in Section 3.2 and Section 3.3, the application of an external magnetic field increased the number of MNPs captured by the nanostructures. Alternatively, reducing the dimensions of the microfluidic channel, particularly the cross-section of the channel, would focus the nanoparticles closer to the nanostructures, where the magnetic field gradient and magnetophoretic force is stronger. The reduction in the microfluidic channel’s cross-section will also reduce the volume of MNP suspension required for these experiments.

Both the total number of captured nanoparticles and capture efficiency were found to be strongly dependent upon the geometry of the ferromagnetic nanostructure. Of the four nanostructure variations, the circular antidot arrays consistently displayed the highest capture efficiencies, while the circular dot arrays consistently displayed the lowest capture efficiencies. Both the circle and square antidot nanostructure variant displayed higher capture efficiencies than their corresponding dot variant, indicating that nanostructures containing antidots will capture most MNPs within their empty spaces than nanostructures comprised of dots. These trends were also observed when applying the MNPs in the presence of both external magnetic fields. This suggests that these trends are a result of the geometries of the nanostructures rather than the alignment of the external magnetic field. Antidot array nanostructures may be useful for biosensing and cell profiling applications in which the nanoparticles, and anything attached to them, need to be captured in predictable locations at regular intervals [14,15,18,23,60]. Conversely, dot nanostructures will capture most MNPs on the outer surface of the dots, between the empty spaces in the nanostructure. If the microfluidic flow was directed into the nanostructure, a dot array nanostructure (without a silicon wafer substrate) could act as a simple and effective filter for the removal or separation of MNPs from a liquid sample [30]. Further research is required to determine the effectiveness of ferromagnetic nanostructure filters in perpendicular microfluidic flows and the capture efficiencies that can be expected in these conditions.

### 4.2. Discussion—MNP Capture with External Magnetic Field Parallel to Nanostructure

The application of the MNPs via microfluidics rather than droplets, under an external magnetic field aligned parallel to the plane of the nanostructured film, noticeably increased the capture efficiencies. This may be due to the microfluidic flow removing nanoparticles that are weakly held by the Permalloy nanostructure. Naturally, the particles caught on the outer surface of the nanostructure are easier to wash away than those nanoparticles caught inside of the nanostructures, which is reflected in the capture efficiencies.

In comparison to the capture of MNPs from the microfluidic flow in the absence of an external magnetic field, the application of a parallel magnetic field has greatly increased the proportion of MNPs captured inside of the nanostructures. This dramatic change in the capture efficiencies was caused by the parallel magnetic field generating attractive magnetophoretic forces over the antidots, as demonstrated in our previous study on the capture of MNPs applied via droplets by ferromagnetic antidot and dot nanostructures [39]. In addition, the total number of MNPs caught by all four nanostructure variants was far greater than in the absence of a magnetic field. The parallel magnetic field strengthened the magnetization of each ferromagnetic nanostructure, which would have produced stronger magnetic field gradients over the nanostructures that penetrate further into the microfluidic channel. The resulting magnetophoretic forces were stronger and directed more MNPs towards the nanostructures.

### 4.3. Discussion—MNP Capture with External Magnetic Field Perpendicular to Nanostructure

After applying nanoparticles to a third set of nanostructures in the presence of a perpendicular external magnetic field, the greatest capture efficiency was once again obtained from the circle antidot nanostructures, indicating that this variation in the nanostructure geometry is best suited for capturing nanoparticles inside the nanostructure, regardless of the external magnetic field. However, the other three geometries exhibit much lower capture efficiencies under a perpendicular magnetic field than in a parallel field. This was expected, since modeling in our previous investigation revealed that the application of a magnetic field perpendicular to an antidot nanostructure results in repulsive magnetophoretic forces above the antidots and attractive forces between antidots [43]. Compared to the results obtained with droplets, the capture efficiencies were considerably higher when employing a microfluidic channel.

The total numbers of magnetic nanoparticles captured by each variation in the nanostructure geometry in the presence of a perpendicular magnetic field were comparable to the numbers obtained in the absence of an external magnetic field. In contrast, far fewer MNPs were captured from the microfluidic flow in the presence of a perpendicular magnetic field than for a parallel magnetic field. Of the two external magnetic field configurations investigated, clearly the greatest number of nanoparticles are captured from the microfluidic flow in the presence of a parallel external magnetic field. Still, the application of a perpendicular magnetic field resulted in a greater number of nanoparticles being captured from the microfluidic flow than in the arrangement without the application of a magnetic field. This suggests that the application of static magnetic fields in any orientation promotes the capture of magnetic nanoparticles by ferromagnetic nanostructures. The total number of nanoparticles captured, and the resulting distribution of nanoparticles caught both inside and outside of the nanostructure, will depend upon the strength and orientation of the external magnetic field.

## 5. Conclusions

We have investigated the capture of 150 nm diameter magnetic nanoparticle clusters by ferromagnetic nanostructures. Four variations in the nanostructure geometry were etched into the surface of three 30 nm thick Permalloy films via focused ion beam lithography. Three microfluidic films, each containing a single straight microchannel, were fabricated from PDMS elastomer using a soft lithographic process. Each microfluidic film was pressed against the surface of a Permalloy film and a 0.1 mg/mL dispersion of MNPs was pumped through each channel. As the MNPs pass over the nanostructures, they are attracted towards and captured by the nanostructures. The resulting distributions of MNPs captured upon and within each nanostructure variant were compared to distributions obtained in a previous study in which MNPs were applied to and captured by ferromagnetic nanostructures while suspended within droplets. As only three sets of nanostructures were fabricated, each experiment could only be performed once for each of the three configurations of the external magnetic field. While the results of these experiments were consistent with our previous experiments on the capture of MNPs by ferromagnetic nanostructures, further trials of each experiment are required to assess the reproducibility of the results presented in this paper. In addition, only two orthogonal configurations of the external magnetic field were investigated. Further trials modifying the orientation of the external magnetic field and nanostructure geometry will lead to a complete understanding of the magnetophoresis and capture of magnetic nanoparticles by ferromagnetic nanostructures. Finally, the capture of MNPs by antidots and dots of varying sizes could also be explored to identify the optimal antidot or dot size for magnetic nanoparticle capture.

The capture efficiencies—the ratio of MNPs captured within the empty spaces of the nanostructure to the MNPs captured on the outer edge of the nanostructure—of all four nanostructure variants were found to be considerably higher when employing a microfluidic channel rather than droplets to apply the MNPs. All four variations in ferromagnetic nanostructure were also found to capture a greater number of nanoparticles in the presence of an external magnetic field. Two configurations of external magnetic fields were investigated, with the field aligned either parallel with or perpendicular to the plane of the nanostructures. The application of a magnetic field parallel to the plane of the nanostructures strongly promoted the capture of MNPs, with the square dot nanostructures recording a 198% increase in total captured MNPs in comparison to capture in the absence of an external magnetic field.

The total number of captured nanoparticles and the capture efficiency were both strongly dependent upon the geometry of the ferromagnetic nanostructure. Of the four variations in nanostructure, the greatest capture efficiency was consistently displayed by the circle antidot arrays, with the majority of captured MNPs—up to 83.1% in a parallel field—captured inside of the antidot holes. The antidot nanostructure variants consistently displayed higher capture efficiencies than the dot variants. Antidot array nanostructures may be particularly useful for biosensing or cell profiling applications. In contrast, the lowest capture efficiency was consistently displayed by the circle dot arrays, with the majority of captured MNPs—up to 84% in a perpendicular field—captured upon the dots rather than inside the nanostructure. Dot array nanostructures may be useful as filters for the removal or separation of MNPs. Future research should investigate the potential application of ferromagnetic nanostructures for the controlled filtration of magnetic nanoparticles from a microfluidic (or potentially macrofluidic) flow.

## Figures and Tables

**Table 1:** The total and average number of magnetic nanoparticles captured from a microfluidic flow by four nanostructure variants in the absence of an external magnetic field.

Nanostructure Geometry	$\mathrm{Total}\;N_{IN}$	$\mathrm{Total}\;N_{OUT}$	$\mathrm{Average}\;N_{IN}$	$\mathrm{Average}\;N_{OUT}$	$\mathrm{Flow}\;C_{EFF}$ (%)	$\mathrm{Droplet}\;C_{EFF}$ (%)
Circle Antidots	152	321	12.88	7.82	62.2	70.8
Circle Dots	7	6	0.19	0.62	23.5	21.3
Square Antidots	50	195	3.13	7.43	29.6	55.9
Square Dots	191	354	7.28	21.07	25.7	35.7

**Table 2:** The total and average number of magnetic nanoparticles captured from a microfluidic flow by four nanostructure variants in the presence of a 138 mT (1.38 kG) external magnetic field directed parallel to the plane of the Permalloy film.

Nanostructure Geometry	$\mathrm{Total}\;N_{IN}$	$\mathrm{Total}\;N_{OUT}$	$\mathrm{Average}\;N_{IN}$	$\mathrm{Average}\;N_{OUT}$	$\mathrm{Flow}\;C_{EFF}$ (%)	$\mathrm{Droplet}\;C_{EFF}$ (%)
Circle Antidots	474	389	46.52	9.48	83.1	70.5
Circle Dots	363	120	9.73	12.43	43.9	44.1
Square Antidots	1095	493	72.04	18.78	79.3	63.9
Square Dots	1204	421	48.16	25.06	65.8	47.7

**Table 3:** The total and average number of magnetic nanoparticles captured from a microfluidic flow by four nanostructure variants in the presence of a 138 mT (1.38 kG) external magnetic field directed perpendicular to the plane of the Permalloy film.

Nanostructure Geometry	$\mathrm{Total}\;N_{IN}$	$\mathrm{Total}\;N_{OUT}$	$\mathrm{Average}\;N_{IN}$	$\mathrm{Average}\;N_{OUT}$	$\mathrm{Flow}\;C_{EFF}$ (%)	$\mathrm{Droplet}\;C_{EFF}$
Circle Antidots	254	277	22.56	6.75	77.0	24.1
Circle Dots	323	400	7.87	41.44	16.0	7.5
Square Antidots	173	268	10.3	10.21	50.2	28.8
Square Dots	206	295	7.85	17.56	30.9	20.4

**Figure A1:**
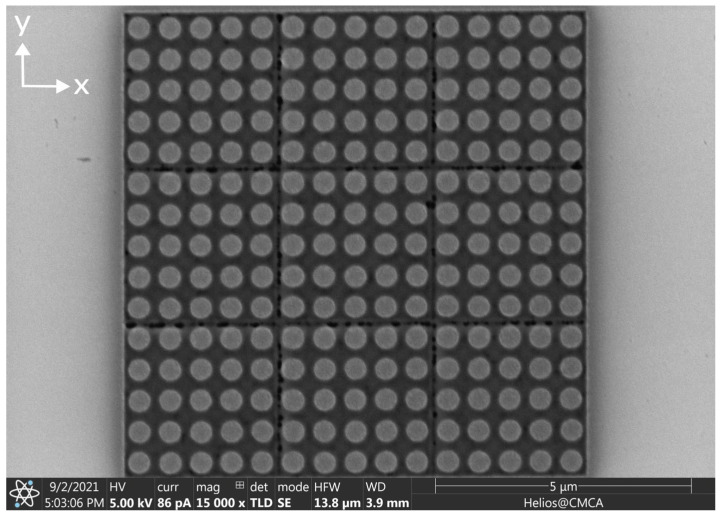
SEM image of a nanostructure consisting of an array of circular dots with 400 nm diameter etched into a 30 nm thick Permalloy film using a focused ion beam. These images were obtained before the application of any MNPs. The centers of the dots are separated by 600 nm in both directions.

**Figure A2:**
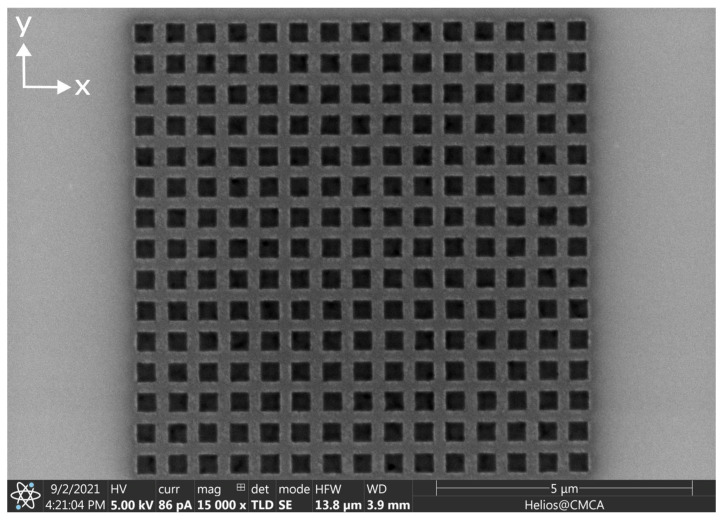
SEM image of a nanostructure consisting of an array of square antidots with sides of length 400 nm etched into a 30 nm thick Permalloy film using a focused ion beam. These images were obtained before the application of any MNPs. The centers of the antidots are separated by 600 nm in both directions.

**Figure A3:**
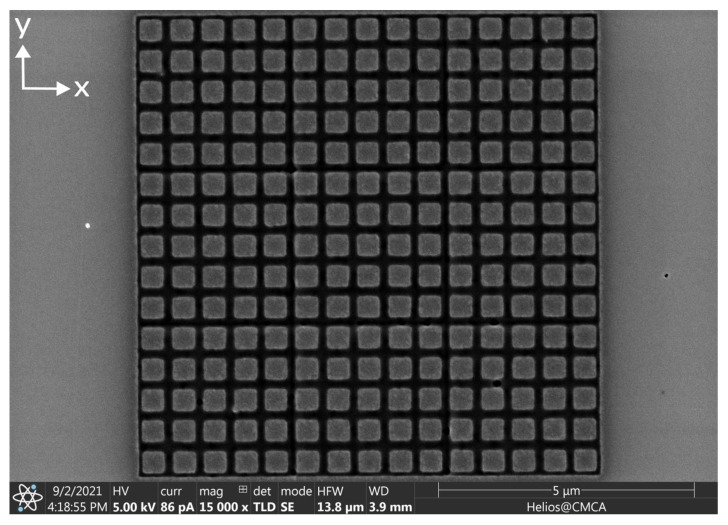
SEM image of a nanostructure consisting of an array of square dots with sides of length 400 nm etched into a 30 nm thick Permalloy film using a focused ion beam. These images were obtained before the application of any MNPs. The centers of the dots are separated by 600 nm in both directions.

**Figure A4:**
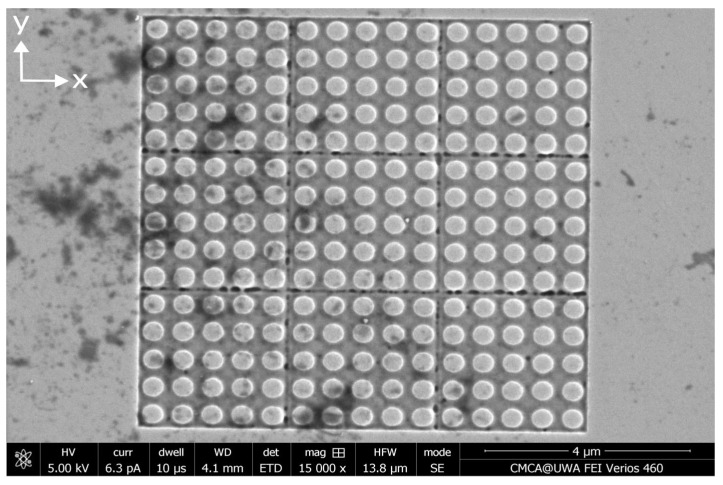
SEM image of MNPs captured by an array of circular dots etched into a thin Permalloy film. The MNPs were applied to this nanostructure via a microfluidic flow in the absence of any external magnetic fields.

**Figure A5:**
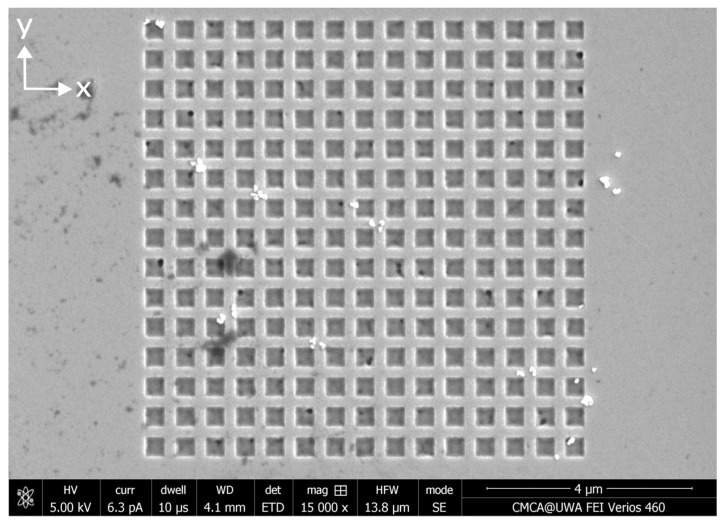
SEM image of MNPs captured by an array of square antidots etched into a thin Permalloy film. The MNPs were applied to this nanostructure via a microfluidic flow in the absence of any external magnetic fields.

**Figure A6:**
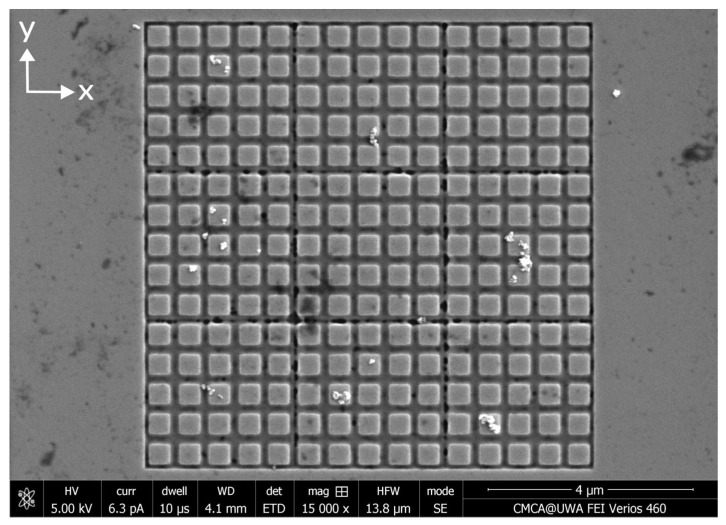
SEM image of MNPs captured by an array of square dots etched into a thin Permalloy film. The MNPs were applied to this nanostructure via a microfluidic flow in the absence of any external magnetic fields.

**Figure A7:**
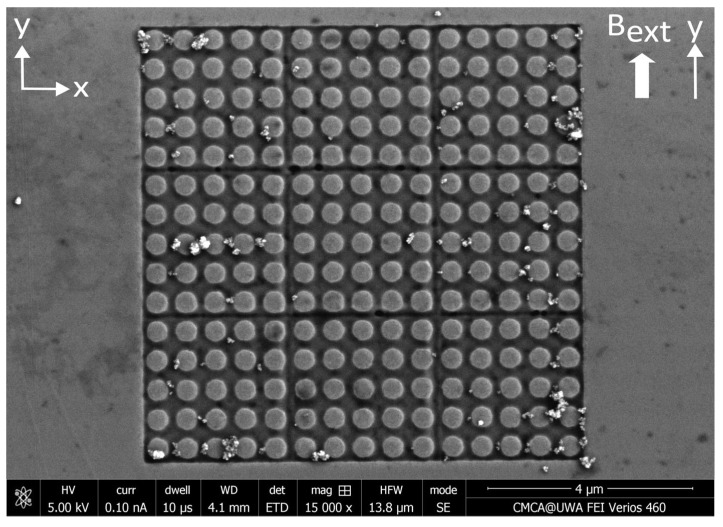
SEM image of MNPs captured by an array of circular dots etched into a thin Permalloy film. The MNPs were applied to this nanostructure via a microfluidic flow under a 138 mT (1.38 kG) static magnetic field applied parallel to the plane of the nanostructures, along the y-axis.

**Figure A8:**
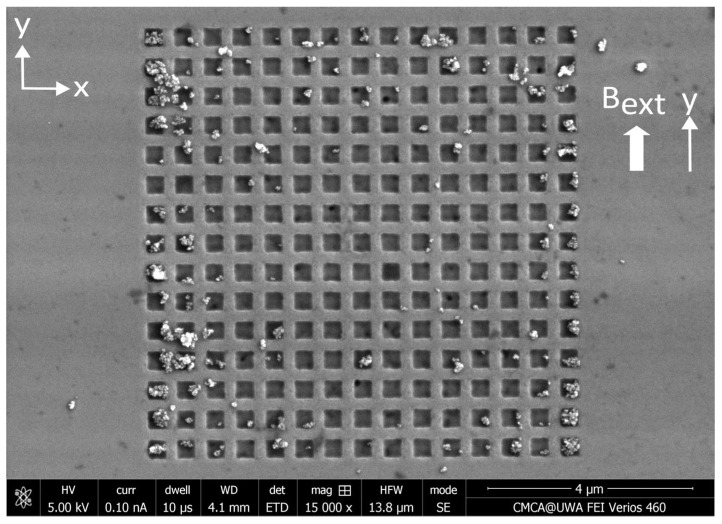
SEM image of MNPs captured by an array of square antidots etched into a thin Permalloy film. The MNPs were applied to this nanostructure via a microfluidic flow under a 138 mT (1.38 kG) static magnetic field applied parallel to the plane of the nanostructures, along the y-axis.

**Figure A9:**
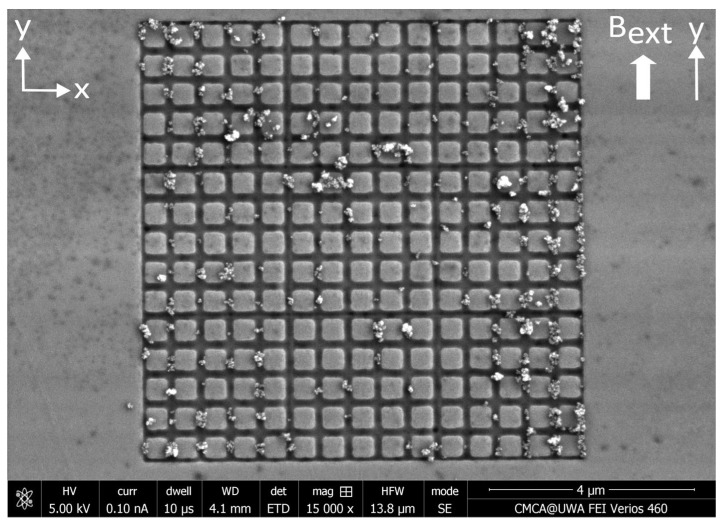
SEM image of MNPs captured by an array of square dots etched into a thin Permalloy film. The MNPs were applied to this nanostructure via a microfluidic flow under a 138 mT (1.38 kG) static magnetic field applied parallel to the plane of the nanostructures, along the y-axis.

**Figure A10:**
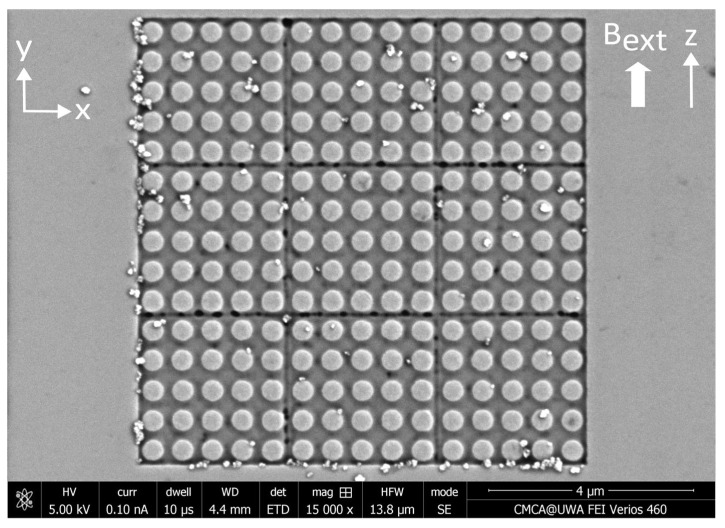
SEM image of MNPs captured by an array of circular dots etched into a thin Permalloy film. The MNPs were applied to this nanostructure via a microfluidic flow under a 138 mT (1.38 kG) static magnetic field applied perpendicular to the plane of the film along the z-axis.

**Figure A11:**
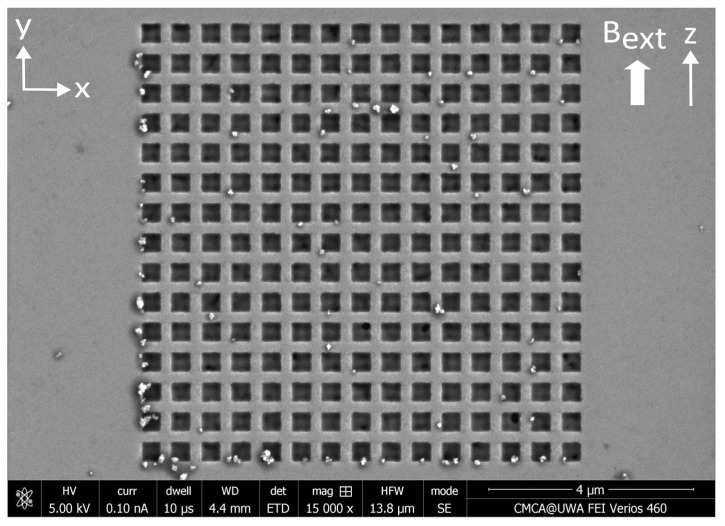
SEM image of MNPs captured by an array of square antidots etched into a thin Permalloy film. The MNPs were applied to this nanostructure via a microfluidic flow under a 138 mT (1.38 kG) static magnetic field applied perpendicular to the plane of the film along the z-axis.

**Figure A12:**
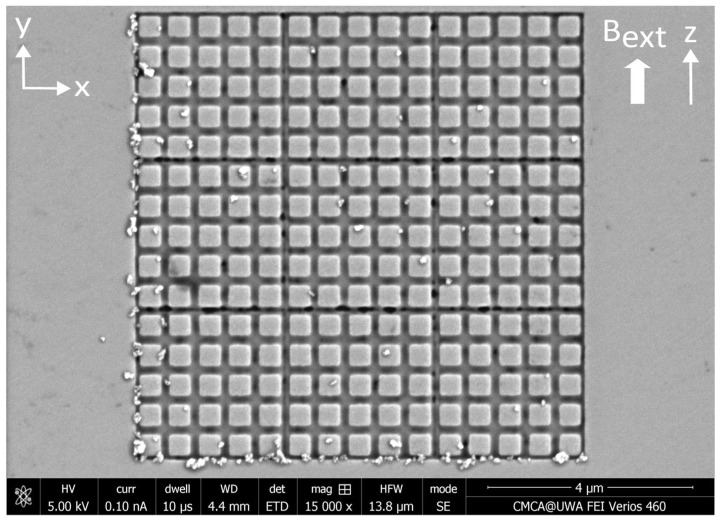
SEM image of MNPs captured by an array of square dots etched into a thin Permalloy film. The MNPs were applied to this nanostructure via a microfluidic flow under a 138 mT (1.38 kG) static magnetic field applied perpendicular to the plane of the film along the z-axis.

**Figure A13:**
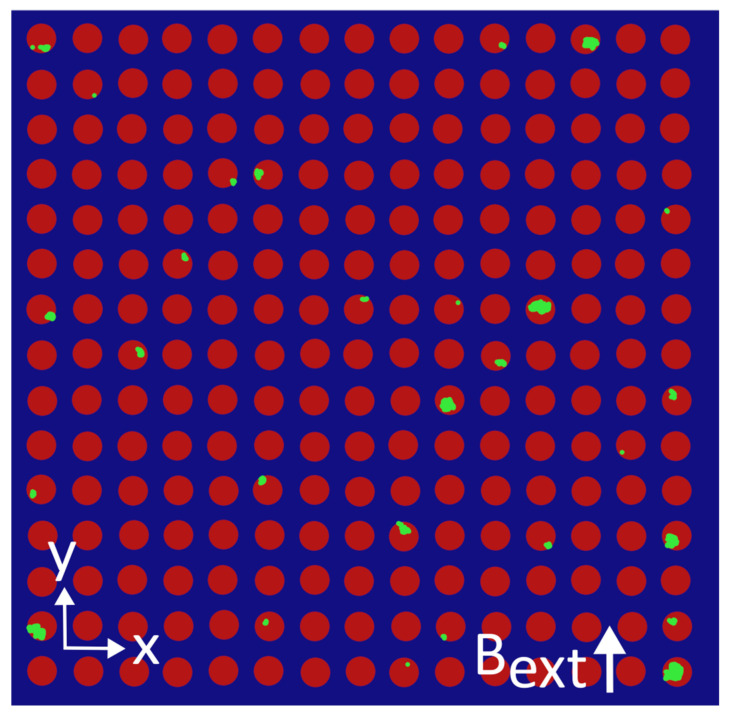
A colorized modification of the SEM image presented in Figure 8, which makes clearer the distribution and sizes of MNPs (green) captured inside the empty space of an array of circular antidots (red) etched into a 30 nm thick Permalloy film (blue). No external magnetic field was present during the capture of these MNPs.

**Figure A14:**
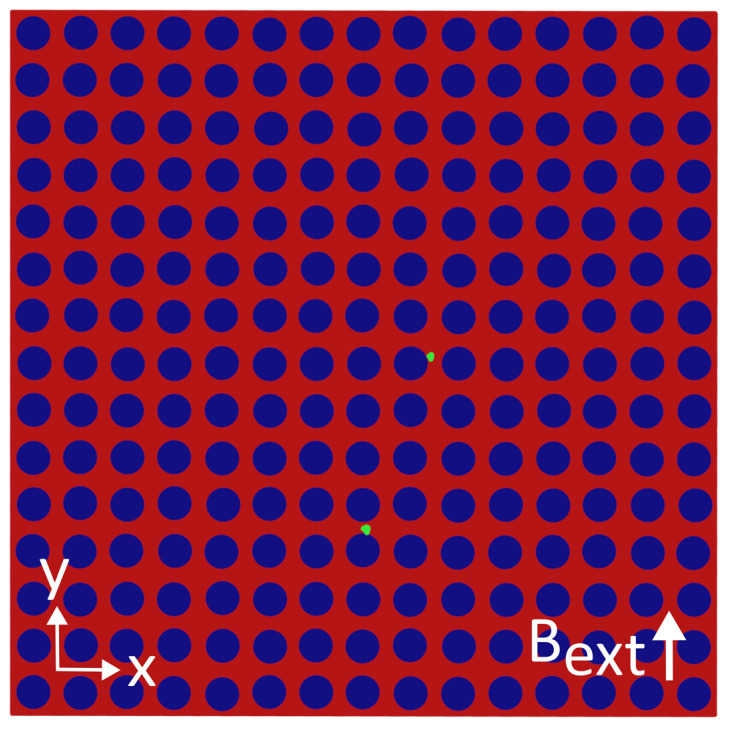
A colorized modification of the SEM image presented in Figure A4, which makes clearer the distribution and sizes of MNPs (green) captured inside the empty space (red) surrounding an array of circular dots (blue) etched into a 30 nm thick Permalloy film. No external magnetic field was present during the capture of these MNPs.

**Figure A15:**
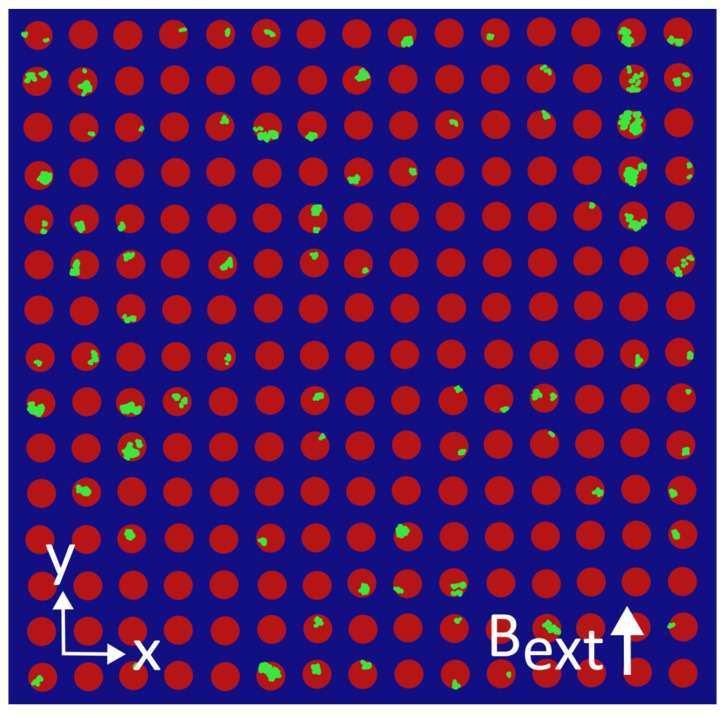
A colorized modification of the SEM image presented in Figure 9, which makes clearer the distribution and sizes of MNPs (green) captured inside the empty space of an array of circular antidots (red) etched into a 30 nm thick Permalloy film (blue). A 138 mT (1.38 kG) external magnetic field parallel to the Permalloy film was present during the capture of these MNPs.

**Figure A16:**
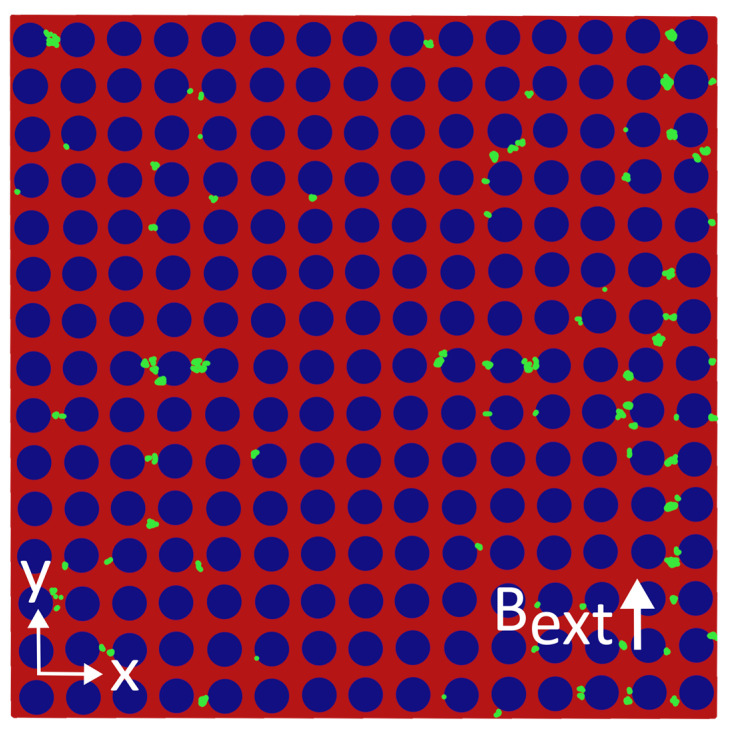
A colorized modification of the SEM image presented in Figure A7, which makes clearer the distribution and sizes of MNPs (green) captured inside the empty space (red) surrounding an array of circular dots (blue) etched into a 30 nm thick Permalloy film. A 138 mT (1.38 kG) external magnetic field parallel to the Permalloy film was present during the capture of these MNPs.

**Figure A17:**
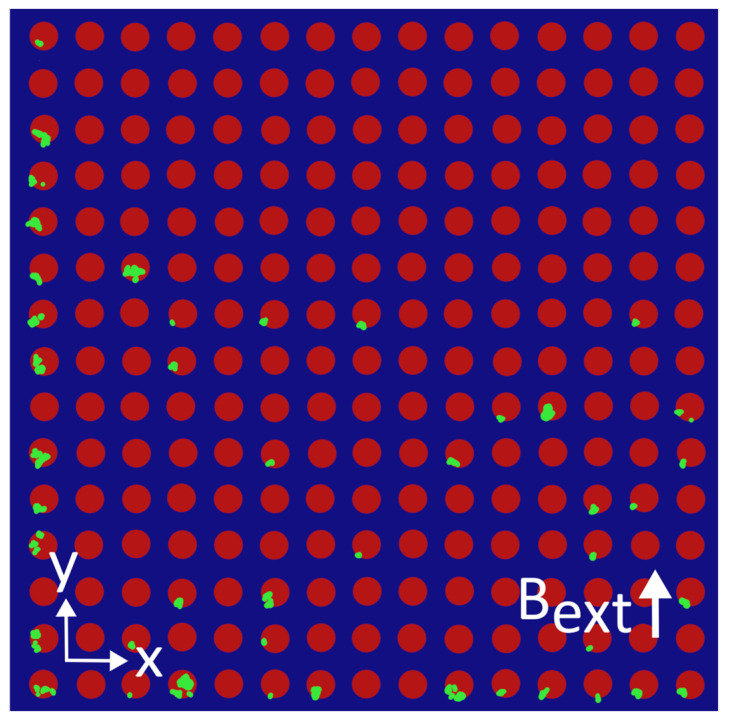
A colorized modification of the SEM image presented in Figure 10, which makes clearer the distribution and sizes of MNPs (green) captured inside the empty space of an array of circular antidots (red) etched into a 30 nm thick Permalloy film (blue). A 138 mT (1.38 kG) external magnetic field perpendicular to the Permalloy film was present during the capture of these MNPs.

**Figure A18:**
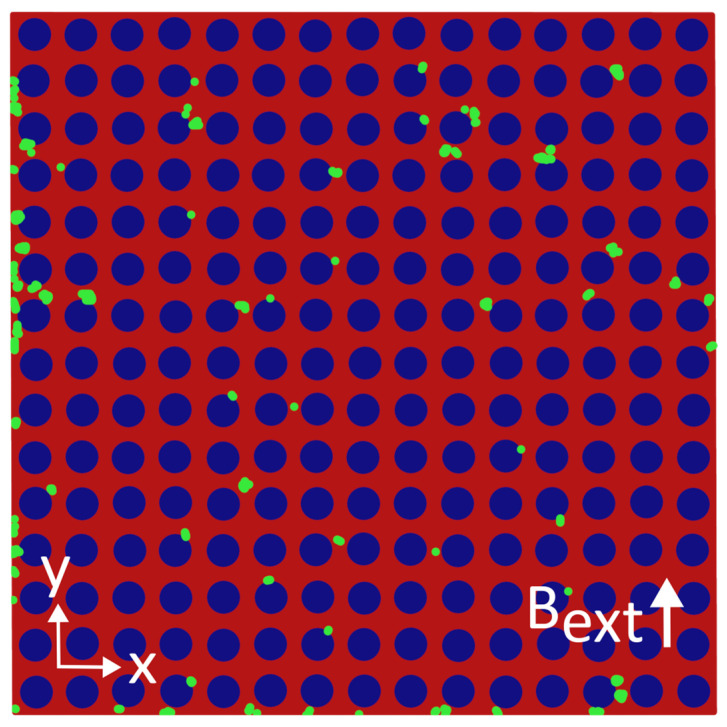
A colorized modification of the SEM image presented in Figure A10, which makes clearer the distribution and sizes of MNPs (green) captured inside the empty space (red) surrounding an array of circular dots (blue) etched into a 30 nm thick Permalloy film. A 138 mT (1.38 kG) external magnetic field perpendicular to the Permalloy film was present during the capture of these MNPs.

## Data Availability

The data presented in this study are openly available in the UWA Profiles and Research Repository at https://doi.org/10.26182/cpdz-fp59.
